# Kir5.1 underlies long-lived subconductance levels in heteromeric Kir4.1/Kir5.1 channels from *Xenopus tropicalis*

**DOI:** 10.1016/j.bbrc.2009.08.032

**Published:** 2009-10-23

**Authors:** Lijun Shang, Sarah V. Ranson, Stephen J. Tucker

**Affiliations:** Clarendon Laboratory, Department of Physics, University of Oxford, Oxford OX1 3PU, United Kingdom

**Keywords:** Kir4.1, Kir4.2, Kir5.1, Potassium channel, Heteromeric channel, Subconductance level

## Abstract

The inwardly-rectifying potassium channel subunit Kir5.1 selectively co-assembles with members of the Kir4.0 subfamily to form novel pH-sensitive heteromeric channels with unique single channel properties. In this study, we have cloned orthologs of Kir4.1 and Kir5.1 from the genome of the amphibian, *Xenopus tropicalis* (Xt). Heteromeric XtKir4.1/XtKir5.1 channels exhibit similar macroscopic current properties to rat Kir4.1/Kir5.1 with a faster time-dependent rate of activation. However, single channel analysis of heteromeric XtKir4.1/XtKir5.1 channels reveals that they have markedly different long-lived, multi-level subconductance states. Furthermore, we demonstrate that the XtKir5.1 subunit is responsible for these prominent subconductance levels. These results are consistent with a model in which the slow transitions between sublevel states represent the movement of individual subunits. These novel channels now provide an excellent model system to determine the structural basis of subconductance levels and contribution of heteromeric pore architecture to this process.

## Introduction

Potassium channels are tetrameric proteins where each of the four subunits contributes to the central K^+^-selective pore. This multimeric architecture permits the co-assembly of different subunits to form novel heteromeric channels with unique functional properties [1,2]. However, it also raises important questions about how the individual movement of these four subunits is translated into opening and closing of a single channel pore.

Of the 80 different K^+^ channel genes found in the human genome, 15 belong to the family of inwardly-rectifying potassium (Kir) channels which are further subdivided into seven different classes (Kir1.1–Kir7.1) [3]. The Kir5.1 subunit does not form functional channels by itself and has no related homologs in the mammalian genome [1,4]. However, Kir5.1 co-assembles with Kir4.1 to form novel heteromeric Kir4.1/Kir5.1 channels which are thought to form the basolateral small conductance K^+^ channel in the distal nephron as well as pH-sensitive K^+^ channels in chemosensitive neurons [1,2]. Likewise, heteromeric Kir4.2/Kir5.1 channels have been reported in hepatic and pancreatic tissues [2,5,6]. The functional properties of heteromeric Kir4.1/Kir5.1 channels are profoundly different to their parental subunits; homomeric Kir4.1 channels are only mildly sensitive to intracellular pH (IC_50_ ∼ 6.0) and have a single channel conductance of approximately 10 pS. By contrast, heteromeric Kir4.1/Kir5.1 channels are highly sensitive to intracellular pH (IC_50_ ∼ 6.8) and have a single channel conductance of ∼45 pS with multiple short-lived, subconductance states [1,2,4,7–10].

The origin of ion channel subconductance states is not well understood [11]. The simplest models of ion channel gating are binary and alternate between two discrete permeation states: open and closed. The movement between these two states is thought to be controlled by a ‘gate’ which physically impedes the flow of ions in the closed state but which moves out of the way during the open state. However, such simple models of channel gating are challenged by the observation of intermediate conductance, or ‘subconductance’ levels, such as those seen in heteromeric Kir4.0/Kir5.1 channels as well as many other types of ion channel [11–14].

Given the tetrameric nature of the K^+^ channel pore it is assumed that the central ion conduction pathway is not formed unless all four of the gating helices are in their ‘open’ conformation. Therefore, one structural model to explain the existence of K^+^ channel subconductance states is that these sublevels originate at the helix-bundle crossing due to successive movements of the four gating helices from the closed to open states, each movement producing a ‘partial’ opening of the channel on the way to the fully open state [11]. An alternative model recently proposed by Chapman & VanDongen suggests that the sublevels seen in the voltage-gated Kv2.1 channel originate from asymmetric conformations adopted by the selectivity filter in response to individual movements of the four gating helices [13]. Either way, both models assume that the allosteric interactions between identical subunits in a homomeric channel are highly cooperative, resulting in rapid transitions between the sublevels which are not resolved in the timescales of most single-channel recordings, making their analysis difficult, especially when obscured by noise and filtering. This behaviour, therefore, gives the appearance of a smooth and binary transition between the open and closed states [11,13].

In this study we demonstrate that the Kir5.1 ortholog from *Xenopus tropicalis* (Xt) produces heteromeric Kir4.1/Kir5.1 channels with very long-lived subconductance states which are readily visible in the recording timescales of conventional patch-clamp electrophysiology. The results support a model in which the observed sublevels relate to the opening movement of individual subunits and provide a new model system which may help provide an insight into the mechanism of K^+^ channel gating and the contribution of heteromeric pore architecture to this process.

## Materials and methods

*Molecular biology.* All channel subunits were subcloned into the oocyte expression vector pBF, which provides 5′ and 3′ untranslated regions from the *Xenopus*-globin gene flanking a polylinker containing multiple restriction sites. In heteromeric Kir channels the subunits were joined in tandem as previously described using a polyglutamine linker [1,2,15]. This method was used in order to control the channel subunit stoichiometry and does not affect channel function. Capped mRNAs were synthesized *in vitro* by using the SP6 mMESSAGE mMACHINE kit (Ambion). Image clones containing the open-reading frames for XtKir4.1 (7660924) and XtKir5.1 (7711180) were ordered from Geneservice (Cambridge, UK).

*Electrophysiology.* Currents were recorded from *Xenopus laevis* oocytes at 22 °C, 18–48 h after injection with XtKir4.1/XtKir5.1. Whole-cell currents recordings were performed with a GeneClamp 500 amplifier. Standard recording solution contained (in mM): 90 KCl, 3 MgCl_2_, 10 Hepes (pH 7.4). Currents were evoked by voltage commands from a holding potential of −10 mV, delivered in 10 mV increments from −120 mV to +40 mV. A well established acetate buffering system which causes intracellular acidification was used to change intracellular pH [2,16]. The acetate solution had the composition: 55 mM KCl, 55 mM potassium acetate, 3 mM MgCl_2_, 2 mM CaCl_2_ and 10 mM Hepes. Single-channel activity was recorded using a Axopatch 200B amplifier in standard cell-attached configuration. The bath and pipette solutions contained the following (in mM): 120 KCl, 1 NaPP, 2 EGTA, and 10 Hepes (pH 7.2). The pipette was filled with (in mM): 120 KCl, 1.8 CaCl_2_, and 10 Hepes (pH 7.2) [17]. Data analysis were carried out with Clampfit 9.2 and Origin 7.0 software and presented as means ± SEM. Results were reproducible at least in three different batches of oocytes.

## Results and discussion

### Identification and cloning of Kir4.1 and Kir5.1 orthologs

**Supplementary Fig. 1:**
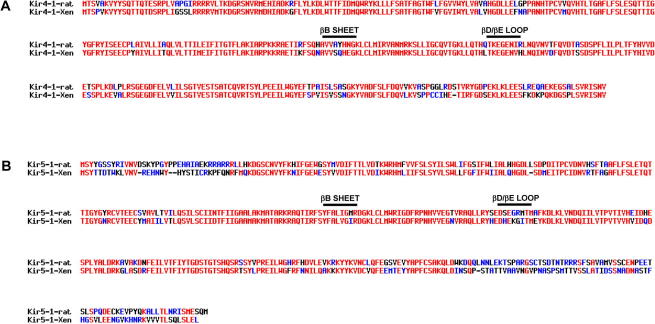
(A) Alignment of Kir4 sequences from rat and *Xenopus tropicalis*. The sequences exhibit 83% sequence identity. (B) Alignment of Kir5.1 sequences from rat and *Xenopus tropicalis*. The sequences exhibit 62% overall identity though this increases to 76% identity if the more divergent distal termini are excluded. The position of the βB sheet and βD/βE loops are marked. The alignment was performed using Multalin (http://bioinfo.genotoul.fr/multalin/) with highly conserved residues being coloured either red or blue according to the degree of conservation (90% and 50%, respectively).

In a previous study we identified a unique heteromeric interaction between the βD/βE cytoplasmic loop of the rat Kir5.1 subunit and the βB sheet of the rat Kir4.1 cytoplasmic domain that influences the gating and rectification of Kir4.1/Kir5.1 channels. The residues involved in this interaction are highly conserved amongst mammalian orthologs of Kir4.1 and Kir5.1 [15]. However, a BLAST search of eukaryotic genomic databases revealed that they are not conserved in the Kir5.1 subunit in the genome of the amphibian, *X. tropicalis*. Analysis of the reported genomic sequences showed that XtKir4.1 shares 83% amino acid identity with rat Kir4.1, but that the XtKir5.1 subunit shows only 62% identity with ratKir5.1, although this increases to 76% if the more divergent distal termini are excluded (Supplementary Fig. 1). Genes encoding the XtKir4.1 and XtKir5.1 open-reading frames were therefore, generated by PCR amplification of appropriate IMAGE clones and subcloned into the pBF expression vector.

### Macroscopic functional properties of XtKir4.1 and XtKir5.1

To study the electrophysiological properties of these novel channels, mRNA was prepared by *in vitro* transcription and injected into *Xenopus* oocytes. In agreement with previous studies of rat Kir5.1, whole-cell currents recorded by two-electrode voltage clamp analysis of oocytes expressing the XtKir5.1 subunit were no different to those found in uninjected oocytes confirming that the XtKir5.1 subunit does not express functional channels by itself. Likewise, oocytes injected with the XtKir4.1 subunit exhibited macroscopic whole-cell currents identical to rat Kir4.1 (not shown). The latter result is consistent with the >90% sequence identity between rat and *Xenopus* Kir4.1.

**Fig. 1:**
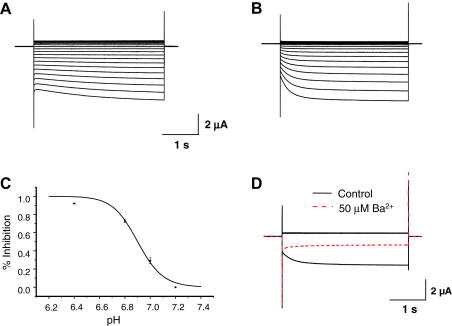
Heteromeric XtKir4.1/XtKir5.1 channels whole-cell currents. Representative whole-cell current traces from (A) rat Kir4.1/Kir5.1 channel and (B) XtKir4.1/XtKir5.1. The strong rectification and time-dependent activation are clearly visible in both cases. However, the Xt channel has a faster time-dependent activation, *τ* = 0.26 ± 0.09 s (*n* = 12) vs. *τ* = 2.01 ± 0.04 s (*n* = 9). (C) Heteromeric XtKir4.1/XtKir5.1 channels also share similar pH sensitivity and (D) Ba^2+^ inhibition properties to the mammalian heteromeric Kir4.1/Kir5.1 channel. The p*K*_a_ of the Xt heteromeric channel, p*K*_a_ = 6.90 ± 0.01 (*n* = 9) is very similar to that of the mammalian channel p*K*_a_ = 6.8 ± 0.1 [8–10]. The currents were evoked by voltage commands from −120 to +40 mV in 10 mV increments, from a holding potential of −10 mV.

In order to study the properties of heteromeric XtKir4.1/XtKir5.1 channels we generated a tandemly-linked dimer where the open-reading frames were fused in tandem by a linker of 10 glutamine residues. This approach has successfully been used to study the electrophysiological properties of rat Kir4.1/Kir5.1 channels as it produces channels of a defined stoichiometry and prevents the formation of homomeric Kir4.1 [1,2,15]. Fig. 1A and B show that oocytes expressing heteromeric XtKir4.1/XtKir5.1 generated inwardly-rectifying K^+^ currents similar to those obtained with rat Kir4.1/Kir5.1, but which showed a significantly faster time-dependent component of activation at hyperpolarizing potentials (XtKir4.1/XtKir5.1 *τ* = 0.26 ± 0.09 s, rat Kir4.1/Kir5.1 *τ* = 2.01 ± 0.04 s). The currents from the tandemly-linked XtKir4.1/XtKir5.1 dimer were also identical to those observed upon coexpression of XtKir4.1 and XtKir5.1 subunits (not shown). Furthermore, similar to the properties of rat Kir4.1/Kir5.1, the XtKir4.1/XtKir5.1 channels were highly sensitive to changes in intracellular pH (p*K*_a_ = 6.9 ± 0.01) (Fig. 1C), and to inhibition by micromolar concentrations of barium (Fig. 1D).

Although the time-dependent component of activation was markedly faster than observed with rat Kir4.1/Kir5.1 this result is consistent with our previous observation that this activation rate is dependent upon the nature of the interaction between the βD/βE cytoplasmic arm of the Kir5.1 subunit and the βB sheet of the Kir4.1 cytoplasmic domain [15]. In this region of XtKir5.1 the residues involved in this interaction differ dramatically thus supporting their role in the control of channel gating (Supplementary Fig. 1).

### Long-lived subconductance states in heteromeric XtKir4.1/XtKir5.1

**Fig. 2:**
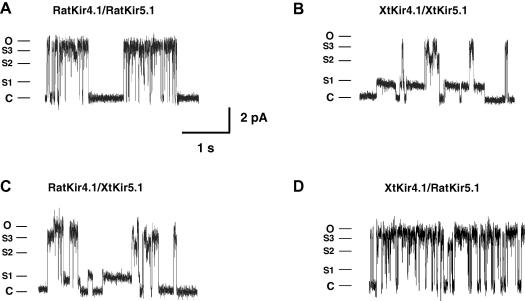
The XtKir5.1 subunit is responsible for the long-lived subconductance levels. Representative traces of single-channel records of (A) ratKir4.1/ratKir5.1 (B) XtKir4.1/XtKir5.1 (C) ratKir4.1/XtKir5.1 and (D) XtKir4.1/ratKir5.1 heteromeric channels. No differences were observed in either the amplitude of the current or the ‘bursting’ single-channel behaviour with multiple subconductance states, but the subconductance states in XtKir4.1/XtKir5.1 heteromeric channels have a much longer duration. In particular, they have a long S1 sublevel opening which is almost 15 times of the average dwell time duration of the S1 sublevels observed in the rat Kir4.1/Kir5.1 channels (7.5 ms vs. 0.5 ms). RatKir4.1/XtKir5.1 heteromeric channels but not XtKir4.1/ratKir5.1 heteromeric channels also exhibit these markedly long S1 sublevels, thus confirming that the XtKir5.1 subunit is responsible for this difference.

Despite the similarity between the macroscopic properties of rat and *Xenopus* Kir4.1/Kir5.1, we observed a profound difference in their behaviour at the single-channel level. Distinctive subconductance levels have previously been observed in rat Kir4.1/Kir5.1 channels but these sublevel states are relatively rare and short-lived [1,2]. An example of single channel currents recorded from rat Kir4.1/Kir5.1 channels is shown in Fig. 2A. By contrast, XtKir4.1/XtKir5.1 channels revealed prominent sublevel states (Fig. 2B). Examination of these sublevels indicates three clearly visible subconductance levels of approximately ¼, ½ and ¾ the size of the fully open level (45 pS) (Figs. 2B and 4A). In particular, they have a long S1 sublevel opening which is almost 15 times the average dwell time duration of the S1 sublevels observed in the rat Kir4.1–Kir5.1 channels (7.5 ms vs. 0.5 ms) (Fig. 2).

### Association of sublevel states with the XtKir5.1 subunit

To determine whether these long-lived subconductance states are generated by the presence of the XtKir5.1, or XtKir4.1 subunit we created a tandemly-linked ratKir4.1/XtKir5.1 dimer. Fig. 2C shows that ratKir4.1/XtKir5.1 heteromeric channels also exhibited markedly long S1 sublevels, but that XtKir4.1/ratKir5.1 channels behaved like rat Kir4.1/Kir5.1 channels (Fig. 2D). This confirms that the presence of the XtKir5.1 subunit within the heteromeric channel is sufficient to generate the long-lived subconductance states.

### Heteromeric Kir4.2/XtKir5.1 channels

**Fig. 3:**
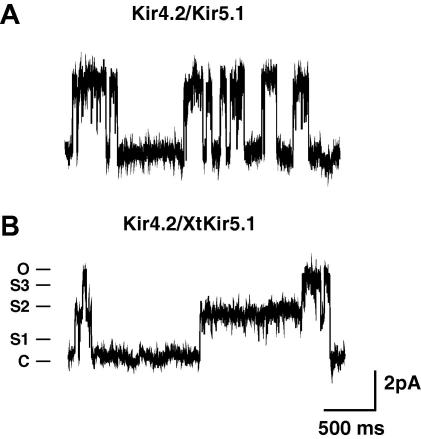
Long-lived subconducatance levels in Heteromeric Kir4.2/XtKir5.1 channels. Representative traces of single-channel records of (A) Kir4.2/Kir5.1 and (B) Kir4.2/XtKir5.1 heteromeric channels. Kir4.2/XtKir5.1 heteromeric channels also exhibited very long-lived subconductance levels as seen in the Kir4.1/XtKir5.1 channels, although in this case the S2 level is the most prominent.

**Fig. 4:**
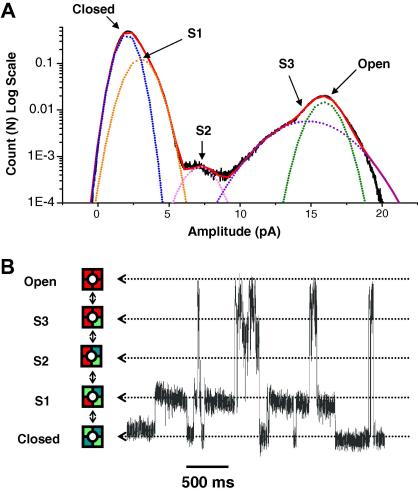
Heteromeric Kir4.0/XtKir5.1 channels as a model system for the study of subconductance levels. (A) Amplitude histogram of heteromeric Kir4.0/XtKir5.1 channels shows the 3 prominent subconductance levels. Arrows point to the peaks of the closed, open and S1–S3 states where individual fits are shown as dotted lines. (B) These sublevels are indicative of the proposed slower transitions of individual subunits to the fully open state in this heteromeric pore. The ability of the XtKir5.1 subunit to slow down the transitions/movements of each subunit in the heteromeric pore makes these channels a suitable model system to study the structural basis of subconductance levels.

In mammals the Kir5.1 subunit can also form novel heteromeric channels with the Kir4.2 subunit [2,5,6]. These channels also exhibit single-channel behaviour similar to the rat Kir4.1/Kir5.1 channel with short-lived subconductance levels visible [2]. However, the *X. tropicalis* genome contains no obvious ortholog of Kir4.2. We therefore, created a tandemly-linked dimer between a rodent Kir4.2 subunit and the XtKir5.1 subunit. Fig. 3 shows that these Kir4.2/XtKir5.1 heteromeric channels also exhibited the very long-lived subconductance levels seen in the Kir4.1/XtKir5.1 channels, although in this case the S2 level was the most prominent. This result further demonstrates the ability of the XtKir5.1 subunit to prolong the duration of these subconductance levels upon heteromultimerisation with Kir4.0 subunits.

### A model system to study movement of K^+^ channel subunits?

The observation of three distinct and prolonged subconductance states in Kir4.0/Kir5.1 heteromeric channels containing the XtKir5.1 subunit is consistent with proposed models in which there is individual movement of all four subunits before the channel enters the fully open state. However, in most multimeric ion channels, and potassium channels in particular, these individual movements normally occur rapidly and beyond the temporal resolution of most single-channel recording methods [13]. Furthermore, the rarity of channel subconductance levels makes their quantitative analysis difficult and time-consuming. A previous study by Xie et al. examined the relationship between binding of the Kir channel agonist PIP_2_ and the subconductance states of Kir2.1. They observed a strong positive cooperativity between subunits promoting the fully open state, but mutant subunits with reduced PIP_2_ affinity were required to observe these sublevel states [18].

We propose that the presence of the XtKir5.1 subunit in heteromeric Kir4.0/XtKir5.1 channels slows down the movement of the subunits within the tetramer sufficiently so that individual gating events can be temporally resolved with conventional single-channel recordings. This therefore, presents a major technical advantage in the ability to record and quantify K^+^ channel subconductance levels. The results are also consistent with the observations made by Chapman and VanDongen that sublevel states are more visible in an asymmetric heteromeric channel [13]. At present we are unable to determine whether these partial openings originate from individual gating motions of the TM2 helix-bundle crossing gates itself or whether, as Chapman and VanDongen propose, they originate from within the selectivity filter itself.

The classical interpretation is that the selectivity filter and the helix-bundle gate are two separate structures at opposite ends of the permeation pathway, where the selectivity filter remains as a rigid body which determines ion selectivity and permeation rate, whilst the constriction at the helix-bundle crossing acts as a dynamic and steric gate. However, although rotational movement of the gating helices and widening of the intracellular vestibule are now almost indisputable during gating, there is increasing evidence that the selectivity filter may also comprise part of the gating mechanism and that there is strong allosteric coupling between this gate and the helix-bundle crossing gate [19–22].

In heteromeric Kir4.1/XtKir5.1 channels, it appears that the transitions between the sublevels are markedly slower and this is either due to differences in the cooperativity of intersubunit interactions found in this heteromeric channel, or slower coupling between the two gates within a single Kir5.1 subunit. The latter is more difficult to address because it is not currently possible to study homomeric Kir5.1 channels. However, future studies may help elucidate the structural basis of the different subconductance behaviour between rat and *Xenopus* Kir 5.1 subunits.

In conclusion, we show the XtKir5.1 subunit defines the unique subconductance levels observed in these heteromeric channels. We also propose that heteromeric Kir4.0/XtKir5.1 channels will provide a useful model system for a detailed dissection of the cooperative movement of individual subunits during channel gating and understanding how a heteromeric pore influences channel opening.
